# Quantitation of the A_2A_ Adenosine Receptor Density in the Striatum of Mice and Pigs with [^18^F]FLUDA by Positron Emission Tomography

**DOI:** 10.3390/ph15050516

**Published:** 2022-04-22

**Authors:** Daniel Gündel, Magali Toussaint, Thu Hang Lai, Winnie Deuther-Conrad, Paul Cumming, Susann Schröder, Rodrigo Teodoro, Rareş-Petru Moldovan, Francisco Pan-Montojo, Bernhard Sattler, Klaus Kopka, Osama Sabri, Peter Brust

**Affiliations:** 1 Department of Neuroradiopharmaceuticals, Institute of Radiopharmaceutical Cancer Research, Helmholtz–Zentrum Dresden–Rossendorf, 04308 Leipzig, Germany; m.toussaint@hzdr.de (M.T.); t.lai@hzdr.de (T.H.L.); w.deuther-conrad@hzdr.de (W.D.-C.); r.teodoro@life-mi.com (R.T.); r.moldovan@hzdr.de (R.-P.M.); k.kopka@hzdr.de (K.K.); p.brust@hzdr.de (P.B.); 2 Department of Research and Development, ROTOP Pharmaka Ltd., 01328 Dresden, Germany; s.schroeder@hzdr.de; 3 Department of Nuclear Medicine, Bern University Hospital, 3010 Bern, Switzerland; paul.cumming@insel.ch; 4 School of Psychology and Counselling, Queensland University of Technology, Brisbane 4000, Australia; 5 Department of Research and Development, Life Molecular Imaging GmbH, 13353 Berlin, Germany; 6 Department of Psychiatry, University Hospital Munich, Ludwig–Maximilians–Universität (LMU) Munich, 80336 Munich, Germany; francisco.pan-montojo@med.uni-muenchen.de; 7 Department of Neurology, University Hospital Munich, Ludwig–Maximilians–Universität (LMU) Munich, 81377 Munich, Germany; 8 Department for Nuclear Medicine, University Hospital Leipzig, 04103 Leipzig, Germany; bernhard.sattler@medizin.uni-leipzig.de (B.S.); osama.sabri@medizin.uni-leipzig.de (O.S.); 9 Faculty of Chemistry and Food Chemistry, School of Science, TU Dresden, 01069 Dresden, Germany; 10 The Lübeck Institute of Experimental Dermatology, University Medical Center Schleswig–Holstein, 23562 Lübeck, Germany

**Keywords:** 7–(3–(4–(2–[^18^F]fluoroethoxy–1,1,2,2–d4)phenyl)propyl)–2–(furan–2–yl)–7H–pyrazolo[4,3–e][1,2,4]triazolo–[1,5–c]pyrimidin–5–amine ([^18^F]FLUDA), A_2A_ adenosine receptor (A_2A_AR), Parkinson’s disease (PD), Huntington’s disease (HD), kinetic analysis, preclinical positron emission tomography (PET), simplified reference tissue model (SRTM)

## Abstract

The cerebral expression of the A_2A_ adenosine receptor (A_2A_AR) is altered in neurodegenerative diseases such as Parkinson’s (PD) and Huntington’s (HD) diseases, making these receptors an attractive diagnostic and therapeutic target. We aimed to further investigate the pharmacokinetic properties in the brain of our recently developed A_2A_AR–specific antagonist radiotracer [^18^F]FLUDA. For this purpose, we retrospectively analysed dynamic PET studies of healthy mice and rotenone–treated mice, and conducted dynamic PET studies with healthy pigs. We performed analysis of mouse brain time–activity curves to calculate the mean residence time (MRT) by non–compartmental analysis, and the binding potential (*BP*_ND_) of [^18^F]FLUDA using the simplified reference tissue model (SRTM). For the pig studies, we performed a Logan graphical analysis to calculate the radiotracer distribution volume (*V*_T_) at baseline and under blocking conditions with tozadenant. The MRT of [^18^F]FLUDA in the striatum of mice was decreased by 30% after treatment with the A_2A_AR antagonist istradefylline. Mouse results showed the highest *BP_ND_* (3.9 to 5.9) in the striatum. SRTM analysis showed a 20% lower A_2A_AR availability in the rotenone–treated mice compared to the control–aged group. Tozadenant treatment significantly decreased the *V*_T_ (14.6 vs. 8.5 mL · g^−1^) and *BP*_ND_ values (1.3 vs. 0.3) in pig striatum. This study confirms the target specificity and a high *BP*_ND_ of [^18^F]FLUDA in the striatum. We conclude that [^18^F]FLUDA is a suitable tool for the non–invasive quantitation of altered A_2A_AR expression in neurodegenerative diseases such as PD and HD, by PET.

## 1. Introduction

Besides being a constituent of nucleic acids, the nucleoside adenosine also represents an important signalling molecule, modulating neurotransmission and physiological processes by activating at least four G–protein–coupled adenosine receptor subtypes: A_1_, A_2A_, A_2B_, and A_3_ [1,2,3]. All these adenosine receptor subtypes are present in the brain, among which the adenosine A_2A_ receptor (A_2A_AR) has the highest expression in the striatum [1]. In that region, the A_2A_AR interacts with dopamine signalling by regulating the output of the extrapyramidal motor system [4]. Striatal A_2A_ARs mainly occur in the medium spiny neurons of the dopamine D2 receptor expressing indirect striatal output pathways projecting to the subthalamic nucleus [5]. A_2A_ARs frequently form heterodimers in complex with other G–protein coupled receptors such as the dopamine D_2_, metabotropic glutamate mGluR_5_, cannabinoid CB_1_, and adenosine A_1_ receptors [6]. Hypoxanthine caffeine is an antagonist of all four adenosine receptor subtypes, with the highest affinity towards the A_2A_AR (*K*_i(human)_ = 9.5–23.4 mM) [7,8], which is thought to mediate its psychostimulant and nootropic effects [9].

The A_2A_ARs modulate GABAergic, glutamatergic, and cholinergic responses in the striatum [10], and an altered receptor expression is implicated in neurodegenerative disorders such as PD [11], as well as HD [12] and Alzheimer’s disease [13]. New treatment strategies in PD seek to potentiate the efficacy of dopamine–replacement therapy by targeting adenosine–dopamine interactions [10], especially in the context of levodopa–induced dyskinesias [14]. Additionally, A_2A_AR antagonist treatment had neuroprotective effects attributed to its anti–inflammatory actions [15,16,17], in contrast to the first–line PD treatment with levodopa [18]. The A_2A_AR antagonist istradefylline (KW–6002, Nouriast^TM^) has recently received FDA approval for adjunctive treatment in patients with PD [19], while a phase III trial with preladenant (SCH 420814) was terminated due to a lack of efficacy [20]. The highly selective A_2A_AR antagonist tozadenant (SYN–115) [21] was well–tolerated in a phase IIb study as a levodopa adjunct in PD patients [22], but was discontinued at phase III because of hematological toxicity [20].

Non–invasive receptor occupancy studies by positron emission tomography (PET) can serve to determine dose–dependent target engagement for optimisation of new medications and to provide non–invasive biomarkers for assessing neuroreceptor changes in PD and other progressive neurodegenerative diseases [23]. A number of PET tracers are available for assessing A_2A_AR availability in the living brain, e.g., [^11^C]KF17837 [10], [^11^C]CSC [11], [^11^C]KF21213, and [^11^C]SCH442416, or the ^18^F–labeled tracers [^18^F]MRS5425/[^18^F]FESCH and [^18^F]MNI–444, of which some suffer from a low signal–to–noise ratio or slow kinetics [24]. We have recently reported that deuteration of the alkyl chain in [^18^F]FLUDA led to improved metabolic stability and negligible cerebral uptake of radiometabolites compared to the isotopologue [^18^F]FESCH in CD–1 mice [25,26].

In the present study, we evaluate the non–displaceable binding potential (*BP*_ND_) of [^18^F]FLUDA in healthy CD–1 mice and investigate its suitability for detecting striatal A_2A_AR changes in a rotenone–induced murine PD model. Aiming towards clinical translation, we also characterise the binding of [^18^F]FLUDA in pigs–a species with similar brain development as humans [27], which also offers a much larger brain size than rodents, thus allowing better quantitation and minimising partial volume effects.

## 2. Results

### 2.1. Non–Compartmental Analysis and Determination of [^18^F]FLUDA BP_ND_ in Healthy CD–1 Mice

First, we retrospectively calculated the kinetic parameters in the mouse brains by non–compartmental analysis of the [^18^F]FLUDA PET time–activity curves (TAC) for vehicle and blocking conditions (tozadenant or istradefylline pre–treatment) from a published data set [25]. As previously described, treatment with istradefylline, but not tozadenant, significantly reduced the area under the curve (AUC) in the murine striatum (Table 1 and Table 2). The pre–treatment with tozadenant was without effect on the kinetic parameters calculated for the target region striatum or for the reference region cerebellum (Table 1). In contrast, the pre–treatment with istradefylline tended to shorten the time–to–peak and to diminish the TAC peak value in the striatum to a level comparable with the values in the cerebellum. Hence, the mean residence time (MRT) was significantly reduced in the striatum (MRT_veh_: 20 ± 2 min vs. MRT_istra_: 14 ± 0 min, *p* < 0.001) (Table 2). In accordance with the observed AUC_0–60 min_ values, pre–administration of istradefylline did not alter the MRTs in the cerebellum, validating its use as a reference region (Table 1 and Table 2).

**Figure 1:**
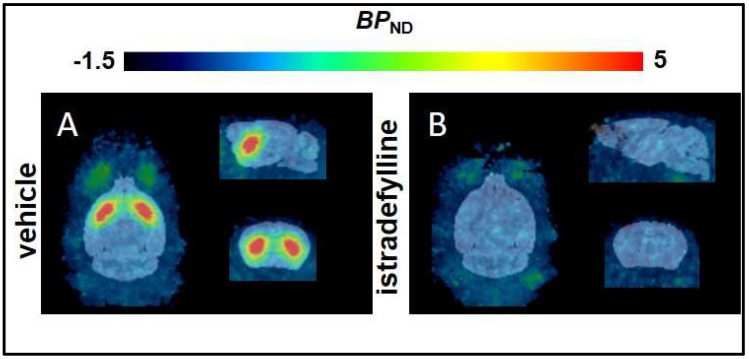
Mean *BP*_ND_ maps derived from simplified reference tissue modelling (SRTM) of [^18^F]FLUDA in the brain of healthy CD–1 mice pre–treated with (**A**) vehicle (*n* = 8) or (**B**) the A_2A_R antagonist istradefylline (1 mg/kg bodyweight, IV, *n* = 4).

Second, we estimated the *BP*_ND_ of [^18^F]FLUDA in the mice by simplified reference tissue modelling (SRTM), which does not require arterial input function. The low affinity of tozadenant towards the murine A_2A_AR (*K*_i_ = 246 nM, [25]) is reflected by the kinetic parameters derived from the brain TACs (Table 1). Therefore, we used the A_2A_AR antagonist istradefylline (*K*_i_ = 58 nM, [25]) as a blocking agent to determine the A_2A_AR–specificity of [^18^F]FLUDA. The mean parametric *BP*_ND_ maps showed high total binding of [^18^F]FLUDA (Figure 1A) in the mouse striatum and a complete blocking by pre–treatment with istradefylline (Figure 1B); the striatal *BP*_ND_ declined from 3.9 ± 1.2 to zero (Table 3). Additionally, the parametric maps did not suggest any displaceable binding in regions other than the striatum.

PET estimates of radiotracer uptake in structures are inherently vulnerable to underestimation due the size of mouse striatum and net spillover of signal. Indeed, the mean *BP*_ND_ values derived from a mouse brain atlas volume of interest (VOI) encompassing the entire mouse striatum (3.9 ± 1.2) were significantly lower compared to findings for a 1 mm spherical VOI (5.9 ± 1.7, *p* < 0.0001), placed within the striatum, centered on the peak activity (Table 3). The R^2^ and Akaike Information Criterion (AIC) provided similar values for the SRTM analyses using either the atlas–based or 1 mm spherical VOIs.

**Table 3:** Striatal [^18^F]FLUDA *BP*_ND_ (SRTM, using the Ma–Benveniste–Mirrione–T2 Atlas whole striatum template, or 1 mm diameter spherical VOI placed in the centroid of the target and reference region of vehicle (veh, *n* = 8) and istradefylline pre–treated (istra, *n* = 4) healthy CD–1 mice.

Brain region		*BP*_ND_			R^2^		AIC	
		Veh	Istra	*p*–Value	Veh	Istra	Veh	Istra
Striatum	T2–Atlas VOI	3.9 ± 1.2	0.0 ± 0.0	*<0.0001*	0.8 ± 0.1	0.8 ± 0.1	100 ± 16	142 ± 22
	1 mm sphere VOI	5.9 ± 1.7	0.1 ± 0.2	*<0.0001*	0.8 ± 0.1	0.7 ± 0.1	106 ± 16	120 ± 16

*p*–value—Student’s *t*–test; R^2^—Spearman correlations; AIC—Akaike information criterion.

### 2.2. Non–Compartmental Analysis and Determination of the BP_ND_ in a C57BL/6JRj Murine Rotenone–Induced Parkinson Disease Model

**Figure 2:**
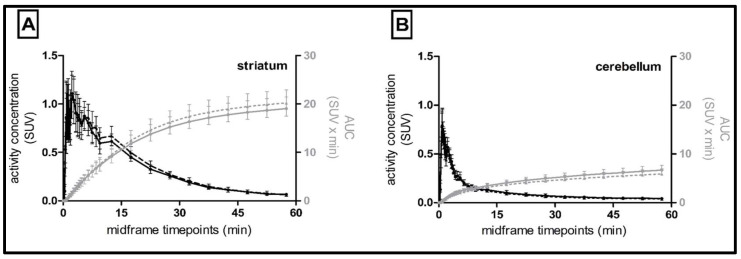
Mean time–activity curves of standardised uptake values (SUV; black lines) and cumulative area under the curves (AUC; grey lines) of control (*n* = 7, dashed lines, triangles) and rotenone–treated (*n* = 6, solid lines, circles) C57BL/6JRj mice. (**A**) striatum and (**B**) cerebellum, mean ± SD.

The [^18^F]FLUDA time–activity curves of the control and rotenone–treated mice were determined retrospectively in the striatum target region and the cerebellum reference region, using the atlas templates. The rotenone–treatment had no significant impact on the radiotracer uptake to the striatum and cerebellum (Figure 2, Table 4). The TAC peak values were observed at an earlier time point in the cerebellum compared to the striatum (0.8 vs. 2.3 min) with lower magnitude (SUV of 0.8 ± 0.1 vs. 1.1 ± 0.2, *p* < 0.001), and lower AUC_0–60 min_ (7 ± 1 vs. 19 ± 2 SUV · min, *p* < 0.001), for both groups as expected for a reference region.

**Figure 3:**
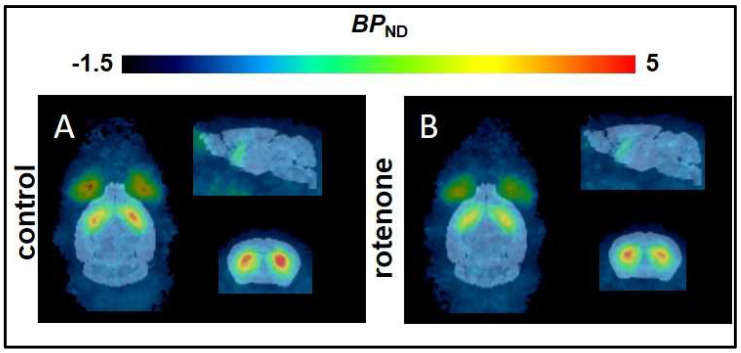
Mean parametric *BP*_ND_ maps derived from SRTM of (**A**) control mice (*n* = 7), and (**B**) rotenone––treated C57BL/6JRj mice (*n* = 6).

Nonetheless, the mean parametric *BP*_ND_ maps suggested a 20% lower striatal *BP*_ND_ in the rotenone–treated mice compared to the control group (Figure 3, Table 5). The R^2^ and AIC were better for the SRTM analysis using the T2–Atlas VOI, although both *BP*_ND_ evaluation strategies showed good agreement. Furthermore, the *BP*_ND_ was lower in the rotenone–treated group regardless of the method of VOI delineation, as suggested also by the TACs (Figure 2, Table 5).

### 2.3. Plasma Metabolism of [^18^F]FLUDA in Pigs

**Figure 4:**
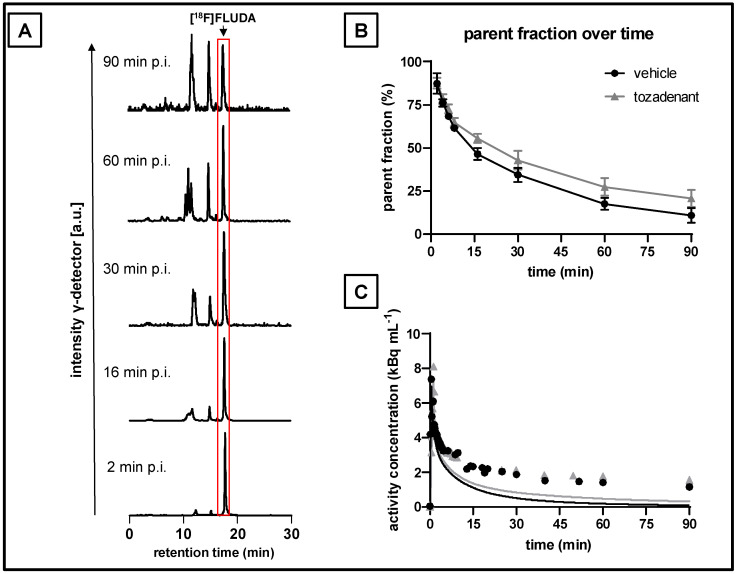
Determination of parent and radiometabolite fractions of [^18^F]FLUDA in plasma samples of pigs and the metabolite–corrected arterial input function. (**A**) Representative RP–HPLC radio–chromatograms of plasma extracts from blood samples collected after IV administration of [^18^F]FLUDA in pigs, (**B**) mean parent fractions in control and tozadenant–treated animals (*n* = 3, mean ± SD), and (**C**) total plasma activity (circles and triangles) and the corresponding metabolite–corrected, bi–exponentially fitted plasma input functions (lines) from representative pigs with (grey) or without (black) tozadenant (bolus + infusion) treatment.

We quantified the parent and radiometabolite fractions for [^18^F]FLUDA in plasma samples of pigs by radio–HPLC. As shown in Figure 4A, we detected up to four different radiometabolites (Figure 4A). As shown in Figure 4B, the parent fraction of [^18^F]FLUDA had declined to 50% at 15 min post–injection in the control pigs, and to 50% at 22 min in the tozadenant group, suggesting competitive inhibition of the enzymatic degradation of the radiotracer by the high plasma concentration of tozadenant. Indeed, the plasma AUC was higher in the tozadenant group (AUC_0–90, tozadenant_ = 82 SUV ∙ min) than in the control group (AUC_0–90_ = 58 SUV ∙ min, vehicle), suggesting a 40% increase in bioavailablility of [^18^F]FLUDA.

### 2.4. Kinetic Analysis of [^18^F]FLUDA Uptake into Different Porcine Brain Regions

**Figure 5:**
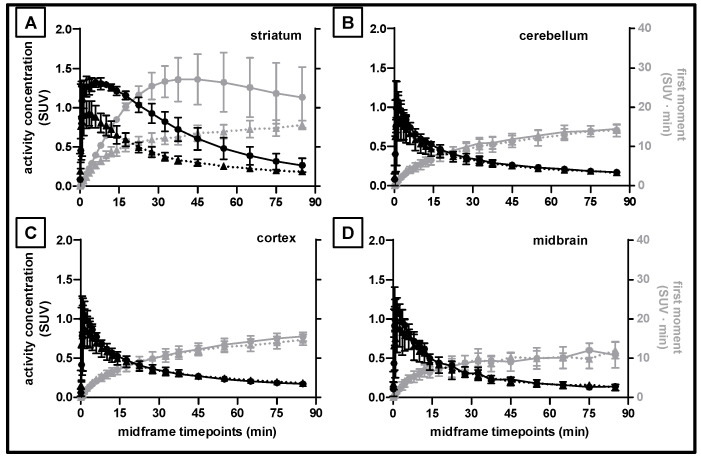
[^18^F]FLUDA mean time–activity curves in standardised uptake values (SUV; black lines) and corresponding first moment curves (grey lines) in different brain regions of pigs with (solid lines, circles) and without (dashed lines, triangles) injection of tozadenant. (**A**) striatum, (**B**) cerebellum, (**C**) cortex, and (**D**) midbrain; *n* = 3, mean ± SD.

We used the standard T1 CH. Malbert pig brain atlas [28] integrated into the PMOD software for the definition of the cerebral subregions. The mean [^18^F]FLUDA TACs for striatum (Figure 5A), cerebellum (Figure 5B), cerebral cortex (Figure 5C), and midbrain (Figure 5D) indicate substantial blockade by pre–treatment with tozadenant only in the striatum. Notably, the cerebellar [^18^F]FLUDA uptake was unaffected by blocking, and thus meets an essential criterion for to serve as a reference region. The same figure also presents the corresponding area–under–the–moment curves (AUMC) used for the calculation of the MRT, along with the other non–compartmental kinetic parameters summarized in Table 6. The TAC peak in the striatum was observed earlier after tozadenant treatment as compared to the control group (1.6 vs. 5.5 min, *p* = 0.05), accompanied by a significantly lower peak TAC value (SUV of 0.9 ± 0.2 vs. 1.3 ± 0.1, *p* = 0.03), and a significantly reduced AUC_0–90 min_ and AUMC_0–90 min_ (*p* = 0.01), all indicating displaceable binding of [^18^F]FLUDA in striatum. These parameters were unaffected by blocking in the other three investigated pig brain regions. Interestingly, the MRT in all brain regions did not differ under control and blocking conditions, indicating that tozadenant treatment did not alter the washout kinetics of [^18^F]FLUDA from the brain.

### 2.5. Determination of the V_T_ and BP_ND_ of [^18^F]FLUDA in the Pig Brain

**Figure 6:**
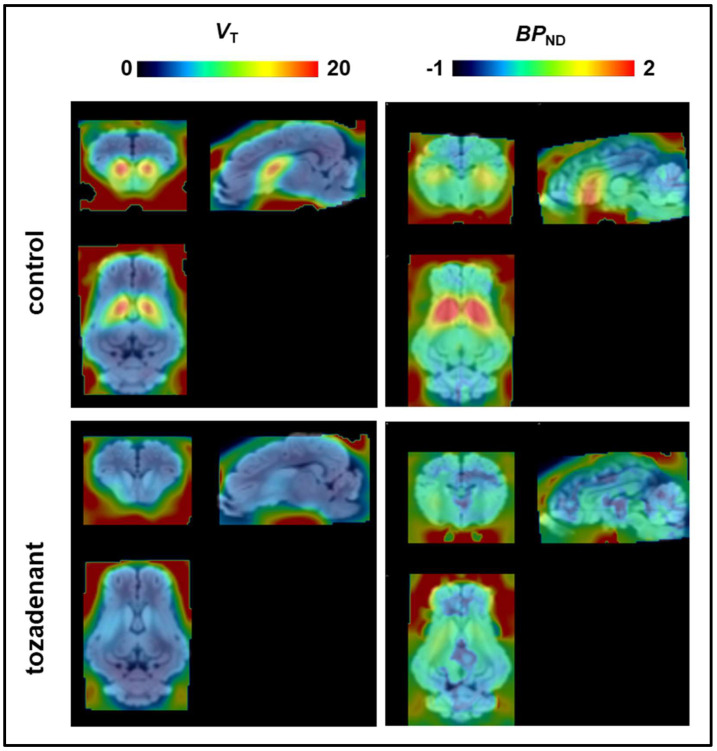
Mean parametric maps of the *V*_T_ (mL · g^−1^) maps (left, *n* = 2) and the corresponding *BP*_ND_ maps (right, *n* = 3) of control and tozadenant–treated pigs.

The mean voxelwise total distribution volume (*V*_T_) maps of [^18^F]FLUDA were calculated by Logan plot analysis and the averaged parametric *BP*_ND_ maps by SRTM (Figure 6). We did not attempt to evaluate the microparameters (*K*_1,_ *k*_2,_ *k*_3,_ and *k*_4)_ because of biased plasma input functions in two animals. However, the compartmental analyses clearly showed complete displacement of the striatal binding of [^18^F]FLUDA by tozadenant treatment. The *V*_T_ maps indicate a global non–specific distribution volume (*V*_D_) of about 7 mL · g^−1^ throughout the blocked pig brain. Notably, the *V*_T_ was two to three–fold higher in the unblocked striatum, while the mean *BP*_ND_ was 1.3 ± 0.4 (1.1 ± 0.3 in nucleus accumbens, 1.4 ± 0.4 in caudate nucleus, and 1.3 ± 0.4 in putamen according to VOI analysis) (Table 7). Tozadenant pretreatment decreased the striatal *BP*_ND_ to 0.31 ± 0.17, corresponding to 76% displacement of [^18^F]FLUDA. The magnitude of *BP*_ND_ was not significantly different from zero in the other two brain regions examined, nor was there clear evidence for displacement by tozadenant.

## 3. Materials and Methods

### 3.1. General Information

All chemicals and reagents were purchased from commercial sources. Tozadenant (toz) was obtained from abcr GmbH (Karlsruhe, Germany), dimethyl sulfoxide (DMSO) and Kolliphor^®^ EL from Sigma–Aldrich (Steinheim, Deutschland), Ursotamin^®^ and physiological sodium chloride (saline) from Serumwerk Bernburg AG (Bernburg, Deutschland), Stresnil^®^ (40 mg/mL) from Elanco Deutschland GmbH (Bad Homburg, Deutschland), Midazolam–ratiopharm^®^ (5 mg/mL), and Heparin–Natrium–25000–ratiopharm^®^ (25.000 IE/mL) from Ratiopharm GmbH (Ulm, Deutschland).

### 3.2. Radiosynthesis of [^18^F]FLUDA

[^18^F]FLUDA was prepared by a two–step one–pot manual (mice studies) or automated (pig studies) radiosynthesis using a ethane–1,2–diyl–*d_4_* bis(4–methylbenzenesulfonate and the corresponding phenol precursor desmethyl SCH442416 (4–(3–(5–amino–2–(furan–2–yl)–7*H*–pyrazolo[4,3–*e*][1,2,4]triazolo[1,5–*c*]pyrimidin–7–yl)propyl)phenol) as previously published [25,29]. Quality control of [^18^F]FLUDA was performed by radio–TLC [silica gel pre–coated plates (Polygram^®^ SIL G/UV_254_, Roth, Germany), eluent mixture: ethyl acetate/petroleum ether 6/1 (*v/v*)] and analytical (radio–)HPLC [ReproSil–Pur 120 C18–AQ column (250 × 4.6 mm, particle size: 5 µm), 10–90–10% MeCN/20 mM NH_4_OAc_aq._*,* flow rate: 1 mL/min]. The radiotracer was obtained with radiochemical yields of 19 ± 3% (manual synthesis, end of bombardment = EOB) or 9 ± 1% (automated synthesis, EOB) and radiochemical purities of ≥ 99%. [^18^F]FLUDA was formulated in an isotonic saline solution (< 10% EtOH *v*/*v* and < 10% DMSO *v*/*v*), which was further diluted into 0.15 mL or 5 mL saline solution for intravenous administration to mice and pigs, respectively.

### 3.3. Animals

All procedures involving animals were performed following national regulations for animal research (Landesdirektion Sachsen, Reg.–Nr.: TVV 18/18; Reference number DD24.1–5131/446/19).

Twelve female CD–1 mice aged 10–12 weeks and weighing 30–35 g were obtained from the Medizinisch–Experimentelles Zentrum (MEZ) at University Leipzig (Leipzig, Germany). Thirteen male mice (control *n* = 7; rotenone–treated *n* = 6) C57BL/6JRj (Janvier Labs, Isle–saint–Genest, France), aged 14 months and weighing 27 to 36 g, were obtained from Pan–Montojo of the Department of Ludwig–Maximilians–Universität (LMU) Munich. The mice were housed with free access to water and food under a 12:12 h dark: light cycle at a constant temperature of 24 °C.

Six pigs (three females and three males) aged six to 12 weeks and weighing 13.6 to 22.6 kg (dams: German Landrace x German Large White, sires: Piétran) were obtained from the Lehr– und Versuchsgut Oberholz (Großpösna, Germany).

### 3.4. Oral Rotenone Administration

Wild–type C57BL/6JRj mice (12 months) were divided into two groups and treated five days a week for two months. A 1.2 mm x 60 mm gavage tube (Unimed, Lausanne, Switzerland) was used to administer 0.01 mL/g bodyweight of rotenone (Sigma–Aldrich, Munich, Germany) solution corresponding to a 5 mg/kg daily dose to the rotenone–treated group (*n* = 6). The control group (*n* = 7) was treated only with the vehicle solution (2% carboxymethyl cellulose (Sigma–Aldrich, Munich, Germany) and 1.25% chloroform (Carl Roth, Karlsruhe, Germany) [26].

### 3.5. Small Animal PET Imaging

The CD–1 mice were divided into three groups: a baseline group (*n* = 8) with intravenous vehicle injection (DMSO/Kolliphor/NaCL, 1:2:7, *v*/*v*) and a pre–treatment group with a blocking agent administered by intravenous injection (istradefylline, 1.0 mg/kg; Bio–Techne GmbH; Wiesbaden–Nordenstadt; Germany or tozadenant, 2.5 mg/kg; abcr GmbH; Karlsruhe; Germany) eight or fifteen minutes prior to radiotracer injection. For the acquisition of the dynamic PET recordings, mice were positioned prone in a custom–made mouse holder (warmed to 37 °C), with the head fixed to a mouthpiece for the administration of 2% isoflurane in 40% air and 60% oxygen (anaesthesia unit: U–410, Agnthos, Lidingö, Sweden; gas blender: MCQ, Rome, Italy) over the whole duration of the PET study. The animals received an injection of [^18^F]FLUDA into a tail vein (3.1–9.7 MBq in 150 µL, 0.7–2.6 nmol/kg, A_m_ at the timepoint of injection: 72–376 GBq/µmol for CD–1 mice; 3.7–8.2 MBq in 150 µL, 1.2–2.8 nmol/kg, A_m_: 81–113 GBq/µmol for C57BL/6JRj mice). We initiated a 60 min PET/MR scan (Mediso nanoScan^®^, Budapest, Hungary) at the time of tracer injection. Subsequently, a T1–weighted gradient–echo sequence (GRE, repetition time = 20 ms, echo time = 6.4 ms) was performed for whole body attenuation correction and anatomical orientation. Additionally, PET data were corrected for random coincidences, dead time, and scatter. The list mode data were sorted into sinograms using a framing scheme of 12 × 10 s, 6 × 30 s, 5 × 60 s, 10 × 300 s. The reconstruction parameters were the following: 3D–ordered subset expectation maximization (OSEM), four iterations, six subsets, energy window = 400–600 keV, coincidence mode = 1–5.

### 3.6. PET Imaging of Pigs

The pigs were initially anaesthetised with intramuscular injections of Stresnil^®^ (0.05 mL/kg bodyweight) and Ursotamin^®^ (0.22 mL/kg bodyweight), and maintained with intravenous administered Ursotamin^®^ and Midazolam–ratiopharm^®^ as required. Additionally, for blood sampling, pigs received an intraperitoneal injection of 0.5 mL Heparin–Natrium–25000–ratiopharm^®^ shortly before starting the PET imaging. Pigs were placed head–first and prone in an ECAT EXACT HR^+^ system (CTI/Siemens) for dynamic PET imaging (90 min; frames: 4 × 15, 4 × 60, 5 × 120, 5 × 300 and 6 × 600 sec). Fifteen minutes before [^18^F]FLUDA administration (123–229 MBq in 5 mL, 0.1–0.2 nmol/kg; A_m_ at the timepoint of injection: 52–186 GBq/µmol), we treated pigs with vehicle (DMSO: Kolliphor^®^ EL: saline in a 1:2:7 composition; *n* = 3) or tozadenant (2.5 mg/kg, followed by continuous infusion of 0.9 mg/kg/h for the duration of the study). The radiotracer and the pharmaceuticals were applied via a catheter placed in the auricular vein.

Reconstruction of the PET scans was done using filtered back projection with a Hanning filter, along with attenuation and further corrections as mandatory (scatter, dead time, decay). A transmission scan with three rotating ^68^Ge rod sources performed prior to the emission scan was used for attenuation correction. After completing the dynamic PET recording, pigs were euthanised with an IV 5 mL dose of T61 (Intervet Deutschland GmbH, Unterschleißheim, Germany).

### 3.7. Blood Sampling of Pigs

The hematocrit was measured in an ear vein blood sample collected just prior to imaging. Blood samples of a volume between 0.5 and 1.0 mL were collected in intervals between 15 and 60 s by a peristaltic pump (P–1, Pharmacia Biotech Inc., Uppsala, Sweden) from a catheter placed in a femoral artery using an autosampler (Fraction Collector FRAC–100, Pharmacia Biotech Inc., Uppsala, Sweden). At circulation times after 40 min, blood samples were drawn by hand every ten min. Subsequently, plasma was obtained by centrifugation (15,000 rpm), and aliquots were counted in a Cobra gamma counter (Packard Instrument Company, Meriden, CT, USA) cross–calibrated to the scanner, and decay corrected for the fluorine–18 half–life. We obtained additional plasma samples for HPLC analysis of radiometabolites at 2, 4, 6, 8, 16, 30, 60 and 90 min post injection (p.i.) of the radiotracer.

### 3.8. Analysis of Radiometabolites

Blood plasma was mixed with the two–fold volume of acetone/water (4/1; *v*/*v*), precipitated proteins were removed by centrifugation, and the supernatants were concentrated and analysed by a semi–preparative RP–HPLC (Reprosil–Pur C18–AQ column (150 × 10 mm; particle size: 10 µm) from Dr. Maisch HPLC GmbH (Ammerbruch; Germany). Elution was obtained with a MeCN/20 mM NH_4_OAc_aq_ gradient (pH 6.8) as follows: 0–5 min 18% MeCN, 5–20 min up to 90% MeCN, 20–22 min 90% MeCN, 22–23 min down to 10% MeCN, 23–30 min isocratic 10% MeCN) at a constant flow rate of 3 mL/min.

### 3.9. Data Analysis and Model Description

Image registration and brain volume of interest (VOI) analysis for mouse experiments were performed with PMOD software (PMOD Technologies LLC, v.4.202, Zurich, Switzerland). The time–activity data are expressed as the mean standardised uptake value (SUV) of the entire VOI. Non–compartmental analysis of achieved time activity curves (TACs) were performed with Microsoft Excel to determine the time–to–peak, the TAC peak value, the area under the curve (AUC):

$${\mathrm{AUC}}_{0-t\left(x\right)}=\int_{0}^{t\left(x\right)}c\left(\mathrm{radioactivity}\right)\times \mathrm{dt}$$

where c (radioactivity) is expressed as standardized uptake value normalized to the body weight in g (SUV), the area–under–the–moment curve (AUMC):

$${\mathrm{AUMC}}_{0-t\left(x\right)}=\int_{0}^{t\left(x\right)}t\;\times \;c\left(\mathrm{radioactivity}\right)\times \mathrm{dt},$$

and the mean residence time (MRT):

$$\mathrm{MRT}=\frac{{\mathrm{AUMC}}_{0-t\left(x\right)}}{{\mathrm{AUC}}_{0-t\left(x\right)}}.$$

**Figure 7:**
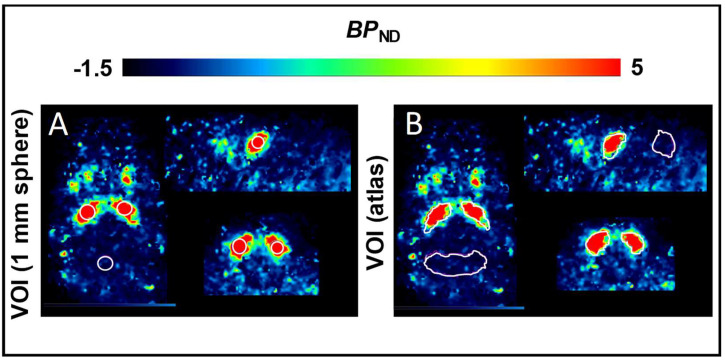
Example of the placement of the volume of interest in mice analysis for (**A**) the 1 mm diameter sphere VOI delineation and (**B**) for the Ma–Benveniste–Mirrione–T2 atlas template delineation of the striatum and cerebellum regions.

Voxelwise maps of [^18^F]FLUDA *BP*_ND_ in the mouse brains were calculated in PMOD by simplified reference tissue model (SRTM) with cerebellum as reference tissue, as previously validated for the related radiotracer [^18^F]FESCH [30] and used as preferred reference region in A_2A_AR PET studies [31,32]. For the evaluations, we compared two VOI delineations of mouse striatum. First, the whole mouse striatum and whole cerebellum VOI from the Ma–Benveniste–Mirrione–T2 atlas template [33], and second, a 1 mm diameter sphere centred on the “hottest” voxels of the left and right striatum left and right and one positioned in the centre of the cerebellum to avoid potential signal spill–in from adjacent structures (Figure 7).

For pig analysis in PMOD, the summed PET brain images were co–registered to the standard T1 C.H. Malbert pig brain atlas [28] and time–activity curves were extracted from the striatum, cerebellum, midbrain and cortex VOIs. The non–compartmental analysis of the pig brain TACs was performed as stated above. Parametric maps of total distribution volumes (*V*_T_; mL ∙ g^−1^) from two control and two tozadenant–treated pigs were calculated by Logan analysis using the metabolite–corrected arterial input function (the plasma curves from the two other scans were corrupted due to technical difficulties during the blood sampling). We calculated the *BP*_ND_ with an SRTM using the cerebellum as a reference region for all six pigs (Table 8). Parametric maps are presented as mean images from two *V*_T_ and three *BP*_ND_ analyses for control and blocking groups.

## 4. Discussion

In the present study, we performed non–compartmental analysis, with additional compartmental analysis to determine the *BP*_ND_ of [^18^F]FLUDA (1) in healthy CD–1 mice, (2) in a rotenone mouse model of PD, and (3) in healthy pigs. We confirmed the A_2A_AR–specific striatal uptake of [^18^F]FLUDA in mice and pigs and the suitability of the cerebellum as a reliable reference region for SRTM analysis. Non–compartmental analysis in the A_2A_AR antagonist–treated animals revealed no impact on the peak time, TAC peak value, MRT, and accumulated activity over time of [^18^F]FLUDA in the reference region, or in any brain regions other than striatum in mice and pigs. In the pig studies, the time–to–peak, TAC peak value, and accumulated activity in the striatum were significantly lower in the group with tozadenant pretreatment, whereas no such effects were detectable in mice with tozadenant pretreatment. However, pre–treatment of mice with istradefylline resulted in significantly lower values for these pharmacokinetic parameters, including a significantly decreased MRT of the tracer in the striatum. The SRTM analysis demonstrated mouse strain and species (mouse vs. pig) differences in the striatal [^18^F]FLUDA *BP*_ND_. Interestingly, we revealed a reduction in *BP*_ND_ in the striatum of the rotenone–treated mice compared to control mice. In pigs, a receptor blockade with tozadenant evoked significantly decreased *V*_T_ and *BP*_ND_ values in the striatum relative to the baseline condition, thus validating the pharmacokinetic results from the non–compartmental analysis.

**Table 8:** A_2A_R receptor affinity (*K*_i_), selectivity, and the striatal binding potential (*BP*_ND_) derived by SRTM (or as stated) of different A_2A_AR-targeting PET radiotracers of different species.

Radiotracer	K_i_ (nM) of ligands	Ratio A_1_/A_2A_	*BP*_ND_ in Striatum and Striatal Substructures	References
[^11^C]TMSX ([^11^C]KF18446)	Rat (forebrain membranes) ^a^: 5.9	Rat: 270	**Human:** 1.5 (DVR + 1)	[34,35]
[^11^C]KW-6002	Human (CHO cells) ^a^: 12/9.1 Rat (synaptosome preparations) ^a^: 2.2/1.6 Mouse (synaptosome preparations) ^a^: 18.9	Human: >31.5 Rat: 32.4 Mouse: 56	**Human:** Caudate 3.4; Putamen 2.9; Nucleus accumbens 2.4	[5,36,37]
[^11^C]SCH442416	Human (CHO cells) ^b^: 0.05 Rat (striatal membranes) ^b^: 0.5	Human: 23145 Rat: 3630	**Macaca nemestrina**: 0.74 **Human:** Caudate 0.53/0.40/0.96 *; Putamen 0.99/0.97/1.67 *	[14,38,39]
[^11^C]Preladenant (SCH 420814)	Human(HEK293 cells) ^b^: 1.1 Rat: 2.5	Human: 343 Rat: 1340	**Wistar rat:** 5.0 to 6.1	[40,41,42]
[^18^F]FPSCH	53.6		**Wistar rat:** 1.4–2.6	[30]
[^18^F]MRS5425/[^18^F]FESCH	Human (HEK293 cells/CHO-K1 cells): 12.4 ^a^/0.6 ^c^	Human: ~ 806/338	**Wistar rat:** 1.6–3.4 **CD-1 mouse:** 2.7–3.8	[26,30,43] Present study
[^18^F]MNI-444	Human (HEK293 cells) ^a^: 2.8		**Macaca mulatta:** Caudate 5.5–6.8; Putamen 8.0–9.6; Nucleus accumbens 2.6–3.5 **Human:** Caudate 2.6–3.6; Putamen 4.1–5.5; Nucleus accumbens 1.3–2.5	[21,44]
[^18^F]FLUDA	Human (CHO-K1 cells): 0.7	Human: >1400	**CD-1 mouse:** 3.9–5.9 **Mouse (C57BL/6JRj, rotenone treated):** 2.5–3.5 **Pigs:** Caudate 1.1–2.1; Putamen 0.9–1.9; Nucleus accumbens: 0.8–1.5	[25] Present study

* *BP*_ND_ in stated brain regions of human subjects: controls/PD/PD with levodopa-induced dyskinesia; *in vitro* displacement of ^a^-[^3^H]CGS21680 (*K*_D_ human A_2A_AR = 22 to 28 nM, *K*_D_ rat A_2A_AR = 14/57 nM, *K*_D_ mouse A_2A_AR = 65 nM, *K*_D_ pig A_2A_AR = 23 nM, agonist [45,46])^, b^-[³H]SCH58261 (*K*_D_ human A_2A_AR = 2.3 nM, antagonist [47]), ^c^-[^3^H]ZM241385 (*K*_D_ human A_2A_AR = 0.23 nM, *K*_D_ rat A_2A_AR = 0.14/0.4 nM [46,48]).

Table 8 shows *BP*_ND_ values in striatum ranging from 0.74 to 9.6 for other radiotracers used for A_2A_AR imaging in different species [5,21,30,49]. The *K*_i_ values of those radiotracers *in vitro* are in the range of 0.05 nM to 12 nM for the human A_2A_AR and 0.5 to 18.9 nM for the A_2A_AR of rodents. Hence, FLUDA possesses a high affinity towards the human A_2A_AR (*K*_i_ = 0.7 nM), as shown by competition assays with [^3^H]ZM241385 [25]. Analyses by the SRTM method have determined the cerebral cortex, midbrain, and cerebellum to serve as reference regions for the calculation of *BP*_ND_ in striatum [21,30,44]. In the present study, we used the cerebellum as a reference region, with the VOI positioned and scaled to avoid significant partial volume effects, even in the small mouse brain. A_2A_AR agonist treatment evoked an increase of cerebral blood flow in rats [49] and the A_2A_AR antagonist tozadenant decreased regional cerebral blood flow in humans [50,51]. While treatment–evoked perfusion changes might conceivably alter [^18^F]FLUDA uptake, we saw no effects of A_2A_AR blockade in the non–compartmental analysis of reference regions in healthy CD–1 mice and pigs. The reductions in the time–to–peak, TAC peak values, and AUCs in the striatum under blocking conditions compared to baseline reflect the A_2A_AR–specific binding in this brain region. The striatal *BP*_ND_ values of [^18^F]FLUDA determined in healthy CD–1 mice under baseline (3.9) and istradefylline blocking condition (0.0) indicate high specificity of the radiotracer towards the A_2A_AR. The apparent magnitude of *BP*_ND_ in the mouse striatum using a 1 mm spherical VOI placed near the centroid of activity (5.9) was considerably higher compared to the *BP*_ND_ estimation by the atlas–based VOI for whole striatum (3.9). This is indicative of the penalty in accuracy due to spillover of signal from the mouse striatum, and may favour the use of a more stringent VOI in rodent PET studies. Similarly, the limited spatial resolution of PET led to systematic underestimation of the true *BP*_ND_ of the D_2_R radiotracer [^18^F]fallypride in the mouse striatum relative to gold standard *ex vivo* determination [52].

[^18^F]FLUDA presents a more favourable *BP*_ND_ (3.9–5.9 in CD–1 mice), compared to its isotopologue [^18^F]FESCH (*BP*_ND_ of 1.6–3.4 in rat striatum [30] vs. 2.7–3.8 in CD–1 mice (data not shown). This difference might reflect methodological factors, or inherent effects of deuteration on the ligand affinity. Furthermore, the enhanced stability of [^18^F]FLUDA (parent fraction of > 99% in the mouse brain at 15 min p.i.) compared to [^18^F]FESCH (parent fraction of 71% [26]) reduces the bias in quantitation due to brain–penetrant radiometabolites, which may be a factor explaining the higher *BP*_ND_ of [^18^F]FLUDA. In the present study, the differing [^18^F]FLUDA *BP*_ND_ between C57BL/6JRj mice and CD–1 mice (2.5 ± 0.4 vs. 3.9 ± 1.2 respectively, p = 0.005) suggests an important effect of strain on A_2A_AR availability *in vivo*. While the two mouse strains also differed with respect to age, clinical PET studies in humans did not indicate important age–dependent changes in A_2A_AR availability [53,54].

Rotenone treatment evokes behavioral parkinsonism and about 75% depletion of striatal dopamine content in rodent [55]. Interestingly, we found reduced striatal A_2A_AR availability in the rotenone model mice as compared to the control–aged group (Table 5). Similarly, Zhou et al. showed a small decrease of striatal *BP*_ND_ with [^11^C]preladenant in 6–OHDA–induced parkinsonian rats compared to sham rats (*BP*_ND_ 4.3 vs. 4.6), suggesting post–synaptic effects of dopamine depletion on A_2A_AR availability [56]. Indeed, a loss of A_2A_AR on striatal medium spiny neurons stands in contrast to the increased expression of dopamine D_2_Rs reported in a model where rotenone was directly administered to the substantia nigra [57], and in postmortem human brain studies [58]. However, Bhattacharjee et al. found elevated striatal uptake of [^18^F]MRS5425 ([^18^F]FESCH) in the 6–OHDA–induced PD model of rats [59]. The inconsistent A_2A_AR PET findings in PD model animals may be due to lack of standardisation in the treatment protocols, and time dependence of the phenotypical changes [60]. Thus, the shorter rotenone treatment of two month in our present study may have induced transient receptor changes, which were not observable in the earlier study with rotenone treatment of four months [26]. Furthermore, A_2A_AR expression on glial cells in the rodent brain may contribute to the PET signal [61,62]. Hence, further investigation is required to establish and explain the effects of rotenenone–induced parkinsonism on striatal A_2A_AR, and the relationship with dopamine D_2_Rs coexpressed on medium spiny neurons.

A_2A_ARs on medium spiny neurons are also implicated in the neurochemical pathology of Huntington’s disease (HD). In autoradiographic studies with [^3^H]CG21680, Martinez–Mir et al. detected a decrease of the A_2A_AR density in the basal ganglia from patients with HD, but that finding *in vitro* has yet to be confirmed using A_2A_AR PET in living HD patients [63]. Thus, PET imaging of A_2A_AR with [^18^F]FLUDA could prove to be a valuable tool for the staging of HD and intervention studies, as seen in pre–clinical models [64,65]. Activation of A_2A_AR on striatal or extrastriatal neurons had opposite effects on psychomotor activity [66]. However, neither [^18^F]FLUDA, nor other available radiotracers, are able to detect the low A_2A_AR density in extrastriatal regions.

In terms of scale, the pig brain presents a distinct advantage over the rodent brain for molecular imaging by PET. On the other hand, the *in vivo* metabolite analysis of [^18^F]FLUDA in pigs revealed faster biotransformation of the radiotracer over time (Figure 4), as compared to CD–1 mice, in which the parent fraction of [^18^F]FLUDA in plasma collected at 15 min p.i. was still 71% [25] vs. only 50% in the pig. Additionally, we have already reported on the formation of at least two additional metabolites in pigs [25]; it remains unknown if the hydrophobic metabolites seen in Figure 4A can cross the blood–brain barrier, thus contributing to brain activity. The non–compartmental analysis did not indicate any effect of tozadenant pretreatment on the striatal [^18^F]FLUDA uptake in CD–1 mice. However, continuous infusion of tozadenant throughout the pig recording resulted in an almost complete displacement of the striatal binding [^18^F]FLUDA. The present estimate of striatal *V*_T_ of [^18^F]FLUDA in pigs (14.6 mL ·g ^−1^, Logan graphical analysis) is comparable to the [^18^F]MNI–444 *V*_T_ in monkeys (12.4–30.3 mL · g^−1^, Logan graphical analysis) [21]. Human striatum shows a regionally heterogeneous distribution of A_2A_ARs, with higher levels in the putamen compared to the head of the caudate nucleus [14,35]. We see some hint of gradients in [^18^F]FLUDA uptake in pig striatum, although less than in a similar sized non–human primate brain (Table 8). Remarkably, the primate and pig results suggest lower *BP*_ND_ than what we estimated in the mouse striatum, despite its small size. This is consistent with the previously determined receptor density *in vitro* with [^18^F]FLUDA in murine (*B*_max_ = 556 ± 143 fmol/mg wet weight) and pig striata (*B*_max_ = 218 fmol/mg wet weight) [25]. In quantitative autoradiographic studies with the A_2A_AR ligand [^3^H]ZM241385, Villar–Menéndez et al. determined a *B*_max_ of 730 fmol/mg protein in putamen of patients dying with PD vs. only 330 fmol/mg protein in controls [58]; we would expect a *BP*_ND_ of [^18^F]FLUDA comparable to our studies in mice. On the other hand, findings of increased A_2A_AR binding in post–mortem brain from PD patients is at odds with our present findings in the acute rotenone model.

## 5. Conclusions

Our study supports the suitability of the SRTM using the cerebellum as a reference region for the evaluation of the *BP*_ND_ of [^18^F]FLUDA in healthy mice, a mouse PD model, and healthy pigs. [^18^F]FLUDA kinetics in pigs differs from that in mice with respect to the greater number and formation rate of plasma radiometabolites, some of which may contribute to brain signals. The magnitude of *BP*_ND_ in the striatum is higher in mice than in pigs, irrespective of the method for quantitation, and despite the greater vulnerability of quantitation in the small mouse striatum to underestimation. However, our investigation in a larger–brained species supports the translatability of [^18^F]FLUDA for the non–invasive PET imaging of A_2A_AR in the human basal ganglia.

## Figures and Tables

**Table 1:** Non–compartmental pharmaokinetic parameters derived from the time–activity curves of [^18^F]FLUDA in target and reference regions in healthy CD–1 mice with vehicle (veh, *n* = 8) or tozadenant (toz, *n* = 4) pre–treatment.

Brain Region	Time–to–Peak (min)			TAC Peak Value (SUV)			AUC_0–60 min_^a^ (SUV ∙ min)			MRT (min)		
	Veh	Toz	*p*–Value	Veh	Toz	*p*–Value	Veh	Toz	*p*–Value	Veh	Toz	*p*–Value
Striatum	3.0 ± 0.8	3.6 ± 2.0	*0.22*	1.0 ± 0.2	0.8 ± 0.1	*0.18*	23 ± 8	18 ± 4	*0.12*	20 ± 2	18 ± 8	*0.30*
Cerebellum	0.9 ± 0.2	1.0 ± 0.3	*0.22*	0.7 ± 0.3	0.7 ± 0.1	*0.49*	6 ± 3	5 ± 1	*0.44*	16 ± 1	15 ± 1	*0.35*
*p*–value *(Striatum* vs. *Cerebellum)*	<0.01	0.02	–	0.05	0.14	–	<0.01	<0.01	–	<0.01	0.24	–

*p*–value—Student’s *t*–test, ^a^ data adapted from [25].

**Table 2:** Non–compartmental pharmaokinetic parameters derived from the time–activity curves of [^18^F]FLUDA in target and reference regions in healthy CD–1 mice with vehicle (veh, *n* = 8) or istradefylline (istra, *n* = 4) pre–treatment.

Brain Region	Time–to–Peak (min)			TAC Peak Value (SUV)			AUC_0–60 min_ (SUV ∙ min)			MRT (min)		
	Veh	Istra	*p*–Value	Veh	Istra	*p*–Value	Veh	Istra	*p*–Value	Veh	Istra	*p*–Value
Striatum	3.0 ± 0.8	1.0 ± 0.3	*<0.01*	1.0 ± 0.2	0.7 ± 0.2	*0.04*	23 ± 8	5 ± 2	*<0.01*	20 ± 2	14 ± 0	*<0.01*
Cerebellum	0.9 ± 0.2	0.9 ± 0.3	*0.32*	0.7 ± 0.3	0.8 ± 0.5	*0.31*	6 ± 3	6 ± 3	*0.36*	16 ± 1	17 ± 2	*0.47*
*p*–value *(Striatum* vs. *Cerebellum)*	<0.01	0.50	–	0.05	0.28	–	<0.01	0.21	–	<0.01	0.02	–

*p*–value—Student’s *t*–test.

**Table 4:** Non–compartmental pharmaokinetic parameters derived from the time–activity curves of [^18^F]FLUDA in target and reference regions in healthy (ctrl, *n* = 7) and rotenone–treated (rot, *n* = 6) C57BL/6JRj mice.

Brain Region	Time–to–Peak (min)		Peak TAC Value (SUV)			AUC_0–60 min_ (SUV min)			MRT (min)		
	Ctrl	Rot	Ctrl	Rot	*p*–Value	Ctrl	Rot	*p*–Value	Ctrl	Rot	*p*–Value
Striatum	2.3	2.3	1.1 ± 0.2	1.1 ± 0.2	*0.91*	20 ± 3	19 ± 2	*0.43*	17 ± 1	17 ± 1	*0.87*
Cerebellum	0.8	0.8	0.7 ± 0.2	0.8 ± 0.1	*0.45*	6 ± 1	7 ± 1	*0.19*	17 ± 1	17 ± 1	*0.59*

*p*–value–Student’s *t*–test.

**Table 5:** Striatal *BP*_ND_ (SRTM) calculated using the Ma–Benveniste–Mirrione–T2 Atlas or use of a 1 mm spherical VOI within the target or reference region and R^2^ for control (*n* = 7) and rotenone–treated (*n* = 6) C57BL/6JRj mice.

Brain Region		*BP*_ND_			R^2^		AIC	
		Ctrl	Rot	*p*–Value	Ctrl	Rot	Ctrl	Rot
Striatum	T2–Atlas VOI	2.5 ± 0.4	2.0 ± 0.4	*<0.001*	0.9 ± 0.1	0.9 ± 0.0	91 ± 16	89 ± 11
	1 mm sphere VOI	3.5 ± 0.7	3.2 ± 0.5	*<0.001*	0.8 ± 0.1	0.8 ± 0.1	121 ± 9	120 ± 13

*p*–value—Student’s *t*–test; R^2^—Spearman correlations; AIC—Akaike information criterion.

**Table 6:** Parameters derived from the non–compartmental analysis of the time–activity curves of [^18^F]FLUDA in different brain regions of pigs treated with (toz) and without (veh) the A_2A_AR specific antagonist tozadenant.

Brain Region	Time–to–Peak Time (min)			TAC Peak Value (SUV)			AUC_0–90 min_ (SUV min)			MRT (min)		
	Veh	Toz	*p*–Value	Veh	Toz	*p*–Value	Veh	Toz	*p*–Value	Veh	Toz	*p*–Value
Striatum	5.5 ± 2.8	1.6 ± 1.7	*0.05*	1.3 ± 0.1	0.9 ± 0.2	*0.03*	61 ± 9	34 ± 5	*0.01*	31 ± 2	30 ± 2	*0.38*
Cerebellum	1.5 ± 0.0	1.0 ± 0.5	*0.43*	1.0 ± 0.2	0.9 ± 0.3	*0.32*	30 ± 2	27 ± 5	*0.27*	31 ± 1	32 ± 2	*0.22*
Midbrain	2.5 ± 1.0	1.3 ± 1.0	*0.11*	1.1 ± 0.1	1.0 ± 0.3	*0.30*	27 ± 2	26 ± 6	*0.35*	27 ± 1	29 ± 2	*0.17*
Cortex	1.5 ± 0.0	1.3 ± 1.0	*0.40*	1.0 ± 0.2	0.9 ± 0.3	*0.29*	30 ± 2	29 ± 5	*0.42*	30 ± 1	32 ± 1	*0.07*

*p*–value—Student’s *t*–test.

**Table 7:** Mean estimates of total distribution volume (*V*_T_; Logan plot) and *BP*_ND_ (SRTM, cerebellum reference region) in pig brain volumes of interest.

Brain Region	*V*_T_ (mL · g^−1^)		*BP*_ND_		
	Veh (*n* = 2)	Toz (*n* = 2)	Veh (*n* = 3)	Toz (*n* = 3)	*p*–Value
Striatum	14.6	8.5	1.32 ± 0.37	0.31 ± 0.17	<0.001
Cerebellum	8.7	7.3	reference	reference	–
Midbrain	6.5	7.0	0.04 ± 0.08	0.05 ± 0.10	0.47
Cortex	7.84	7.90	0.08 ± 0.10	0.1 ± 0.04	0.37

*p*–value—Student’s *t*–test.

## Data Availability

Data is contained within the article.
